# Arnica Hair Wash

**Published:** 1862-07

**Authors:** 


Arnica Hair Wash.—℞ Elder water, half a pint; sherry wine, half a pint; tincture of arnica, half an ounce; alcoholic ammonia, one drachm. Mix in a lotion bottle, and apply to the head every night with a sponge. Wash the head with warm water twice a week. This will be found of great service, when the hair is falling off and becoming thin from too frequent use of castor, macassar oil, etc., or when premature baldness arises from illness.—*Druggist.*

